# Cilostazol Add-On Therapy in Patients with Mild Dementia Receiving Donepezil: A Retrospective Study

**DOI:** 10.1371/journal.pone.0089516

**Published:** 2014-02-26

**Authors:** Masafumi Ihara, Madoka Nishino, Akihiko Taguchi, Yumi Yamamoto, Yorito Hattori, Satoshi Saito, Yukako Takahashi, Masahiro Tsuji, Yukiko Kasahara, Yu Takata, Masahiro Okada

**Affiliations:** 1 Department of Stroke and Cerebrovascular Diseases, National Cerebral and Cardiovascular Center Hospital, Osaka, Japan; 2 Department of Regenerative Medicine and Tissue Engineering, National Cerebral and Cardiovascular Center Research Institute, National Cerebral and Cardiovascular Center, Osaka, Japan; 3 Department of Neurosurgery, Sumoto Itsuki Hospital, Hyogo, Japan; 4 Department of Regenerative Medicine and Research, Institute of Biomedical Research and Innovation, Hyogo, Japan; Oregon Health & Science University, United States of America

## Abstract

**Goal:**

Combinatorial therapy directed at both vascular and neurodegenerative aspects of dementia may offer a promising strategy for treatment of dementia, which often has a multifactorial basis in the elderly. We investigated whether the phosphodiesterase III inhibitor cilostazol, which is often used in the prevention of stroke and peripheral artery disease, may delay cognitive decline in the elderly receiving donepezil.

**Methods:**

Medical records were retrospectively surveyed to identify patients who had received donepezil for more than one year and had undergone Mini-Mental State Examination (MMSE) at least at two time points. Those with an initial MMSE score of less than 27 points were subjected to analysis (n = 156), with a cut-point of 21/22 applied to assign them to mild (n = 70) and moderate/severe (n = 86) dementia. The change of total MMSE score per year was compared between patients who had received donepezil and those given both donepezil and cilostazol.

**Findings:**

In patients with mild dementia who had received donepezil and cilostazol (n = 34; 77.2±6.8 years old), the annual change in MMSE score was −0.5±1.6 during an observational period of 28.6±11.7 months, with those receiving donepezil only (n = 36; 78.4±6.5 years old) scoring less (−2.2±4.1) during 30.4±12.8 months with a statistical intergroup difference (p = 0.022). Multivariate analysis showed that absence of cilostazol treatment was the only significant predictor of MMSE decline. A positive effect of cilostazol was found in three subscale scores of MMSE, orientation for time or place and delayed recall. By clear contrast, in patients with moderate/severe dementia, there were no intergroup differences in decrease of total or subscale MMSE scores between the two groups.

**Conclusions:**

These results suggest potential for cilostazol treatment in the suppression of cognitive decline in patients receiving donepezil with mild dementia but not in those with moderate/severe dementia.

## Introduction

Epidemiological, clinicopathological and animal studies show that vascular disease in various forms contributes to cognitive decline [1], [2]. Increasing age is the strongest risk for dementia irrespective of whether it results from a vascular etiology or neurodegenerative disease processes such as in Alzheimer's disease (AD). AD and vascular cognitive impairment (VCI), the two most common causes of dementia, represent two extremes of a spectrum of disorders; however, a number of entities, which possess varying degrees of neurodegenerative and vascular pathologies, occur in between [3]. The pure forms of the disorders are preferred for convenience to label, treat or manage but conditions within the spectrum are the norm rather than the exception as dementia advances [4]. Therefore, combinatorial therapy directed at both vascular and neurodegenerative aspects of dementia may be a promising approach for the treatment of dementia in the elderly.

Cilostazol acts as an antiplatelet agent and has other pleiotropic effects based on phosphodiesterase-3-dependent mechanisms [5]. Increasing evidence suggests that cilostazol offers endothelial protection, via an inhibition of apoptosis in endothelial cells [6], attenuates the phenotypic modulation of vascular smooth muscle cells [7], and sustains blood flow by endothelium-independent vasodilation [8]. Intriguingly, cilostazol has been shown to decrease amyloid β (Aβ) accumulation and protect Aβ-induced cognitive deficits in an experimental model [9], [10]. In a pilot study for 10 patients with moderate Alzheimer's disease (mean Mini-Mental Examination (MMSE) score, 11.9 points) who received donepezil, cilostazol add-on treatment for 5–6 months demonstrated significantly increased MMSE score in comparison to baseline [11]. Moreover, cilostazol was shown to be effective in halting cognitive decline in patients with AD with cerebrovascular diseases [12] and mild cognitive impairment [13].

From the above experimental and clinical findings, we hypothesized that cilostazol has a potential to delay the cognitive decline through the pleiotropic effects in the demented elderly. We therefore retrospectively surveyed patients with dementia receiving donepezil and determined how cilostazol add-on treatment affected interval change of MMSE.

## Methods

### Subjects

The protocol for this study was designed in accordance with the ethical guidelines for epidemiology study established by Japan's Ministry of Education, Culture, Sports, Science and Technology in December 2008. Because this study examined only preexisting data, written informed consent was not obtained from each patient. However, we publicized the study by posting a summary of the protocol (with an easily understood description) in the Department of Neurosurgery's waiting room at Sumotoitsuki Hospital (Sumoto City, Japan); the notice clearly informed patients of their right to refuse enrolment. These procedures for informed consent and enrolment are in accordance with the detailed regulations regarding informed consent described in the guidelines, and this study, including the procedure for enrolment, has been approved by the Institutional Review Board of Sumotoitsuki Hospital. The electronic medical chart of outpatients, obtained from September 1st 1996 to June 30th 2012, was surveyed to identify cases that had history of administration of donepezil with or without cilostazol and scored MMSE at the interval of more than 1 year.

By focusing on patients whose physicians believed their clinical status was potentially ameliorated by cilostazol, we hoped to gain some degree of comparability in baseline patient characteristics between the donepezil alone and cilostazol add-on groups. To evaluate atrophy of the hippocampus, Z-scores of the voxel-based specific regional analysis system for Alzheimer's disease (VSRAD) were calculated in patients who had magnetic resonance imaging within 30 days of their initial MMSE evaluation [14]. MMSE score was evaluated mainly for purposes of applying for nursing insurance and was not necessarily linked to the date of cilostazol intake.

MMSE was scored by a single examiner who was blind to medication details. Patients who had taken cilostazol for at least 6 months during the intervals of MMSE were enrolled in the cilostazol-treated group. When MMSE was scored more than twice, the first and the last scores were selected. First, change ratio of MMSE score (point/year) was calculated according to (change in MMSE score)/([days of interval of MMSE]/365), as intervals of MMSE significantly varied between patients. Second, enrolled patients were sub-grouped based on the initial score of MMSE. Patients with an MMSE score of 22–26 were defined as mild dementia and less than 21 were moderate/severe dementia. Third, the change in each domain of MMSE score was analyzed to identify the effect of cilostazol *per se*. Hypertension, hyperlipidemia, and diabetes mellitus were defined based on the need for oral anti-hypertensive, anti-hyperlipidemic, or anti-diabetic drug therapy (or insulin), respectively, prescribed by the physician. The intake of drugs that might influence cognitive function such as angiotensin converting-enzyme inhibitor, angiotensin receptor blocker and statin (3-hydroxy-3-methylglutaryl coenzyme A reductase inhibitor) was surveyed in enrolled patients.

### Statistical analysis

For statistical analysis, JMP version 9J was used. Individual comparisons were performed using a chi-square test or 2-tailed, unpaired Student's t-test. Multivariate analysis was performed to determine the effects of several variables such as age, gender, vascular risk factors and medications, including cilostazol, on the annual change in MMSE. Mean ± standard deviation is shown unless stated otherwise. Significance was assumed at p<0.05.

## Results

A total of 3183 patients had a history of cilostazol administration, including 1942 patients who took donepezil as an anti-dementia drug. With the inclusion criteria described above, we initially identified 51 patients with moderate/severe dementia (MMSE score less than 22) treated with donepezil alone (17 men and 34 women; mean 78.2 years old) and 35 patients treated with donepezil plus cilostazol (14 men and 21 women; mean 79.3 years old) (Table 1). The average daily dose of cilostazol was 139 mg/day. There was no significant difference between the donepezil group and the cilostazol add-on group in the initial MMSE score (16.5±4.8 vs. 15.9±4.2; p = 0.51) but there was a trend for significance in the observational period (30.2±11.9 months vs. 25.8±10.2 months; p = 0.08). The duration of cilostazol intake for cilostazol add-on group was 22.4±9.8 months. The annual change of MMSE was −0.9±2.6 in the donepezil group and −0.7±2.8 in the cilostazol add-on group, with no intergroup statistical difference (p = 0.72). The annual change for all 11 subscale MMSE scores was also not significantly different between the two groups. Multivariate analysis did not show any significant predictors of MMSE decline among the following eight explanatory variables: age, gender, initial MMSE score, hypertension, hyperlipidemia, diabetes, cilostazol, and z score of VSRAD. Addition of other explanatory variables such as antihypertensive medications did not affect the result.

**Table 1:** Comparisons of clinical profile and changes in MMSE scores in patients with moderate/severe dementia receiving donepezil (donepezil group) and donepezil plus cilostazol (combination group).

	Donepezil	Combination (donepezil/cilostazol)	p value
Number of subjects	51	35	
Sex (male/female)	17/34	14/21	0.53
Age (years)	78.2±10.5	79.3±5.9	0.58
Initial MMSE	16.5±4.8	15.9±4.2	0.51
Z-scores of VSRAD	2.82±1.57	3.13±1.40	0.39
Observational period (months)	30.2±11.9	25.8±10.2	0.08
Vascular risk factors			
Hypertension	9	10	0.23
Diabetes	6	2	0.34
Hyperlipidemia	4	5	0.34
Treatment, n			
Ca^2+^ blocker	6	6	0.48
ACE inhibitor	2	1	0.79
Diuretics	2	2	0.70
α and/or β blocker	1	2	0.35
ΔMMSE	−0.9±2.6	−0.7±2.8	0.72

MMSE, Mini-Mental State Examination; VSRAD, Voxel-Based Specific Regional Analysis System for Alzheimer's Disease; ACE, angiotensin-converting enzyme. ΔMMSE indicates the changes in MMSE scores [(follow up MMSE)–(initial MMSE)] per year. *p<0.05 in donepezil group vs. combination group.

We identified a further 36 patients with mild dementia (22≤MMSE≤26) treated with donepezil alone and 34 patients treated with donepezil plus cilostazol (Table 2). There were no significant differences in the initial MMSE scores (24.0±1.3 vs. 24.2±1.5; p = 0.43) or the observational period (30.4±12.8 vs. 28.6±11.7; p = 0.52) between the donepezil and the cilostazol add-on groups but there was a trend for significance in the degree of hippocampal atrophy assessed with the VSRAD when the initial MMSE was evaluated (2.44±1.24 vs. 1.92±1.09; p = 0.08). The frequency of cardiovascular risk factors including hypertension, diabetes mellitus, and hyperlipidemia was not different between the two groups but the frequency of treatment for the risk factors was significantly higher in the donepezil alone group for Ca^2+^ blockers (p = 0.04) and diuretics (p = 0.02) compared to the cilostazol add-on group. The average daily dose of cilostazol was 121 mg/day.

**Table 2:** Comparisons of clinical profile and changes in MMSE scores in patients with mild dementia receiving donepezil (donepezil group) and donepezil plus cilostazol (combination group).

	Donepezil	Combination (donepezil/cilostazol)	p value
Number of subjects	36	34	
Sex (male/female)	16/20	15/19	0.97
Age (years)	78.4±6.5	77.2±6.8	0.46
Initial MMSE	24.0±1.3	24.2±1.5	0.43
Z-scores of VSRAD	2.44±1.24	1.92±1.09	0.08
Observational period (months)	30.4±12.8	28.6±11.7	0.52
Vascular risk factors			
Hypertension	15	9	0.18
Diabetes	3	1	0.33
Hyperlipidemia	6	5	0.82
Treatment, n			
Ca^2+^ blocker	13	5	0.04
ACE inhibitor	2	1	0.59
Diuretics	5	0	0.02
α and/or β blocker	3	0	0.09
ΔMMSE	−2.2±4.1	−0.5±1.6	0.02

MMSE, Mini-Mental State Examination; VSRAD, Voxel-Based Specific Regional Analysis System for Alzheimer's Disease; ACE, angiotensin-converting enzyme. ΔMMSE indicates the changes in MMSE scores [(follow up MMSE)–(initial MMSE)] per year. *p<0.05 in donepezil group vs. combination group.

**Figure 1:**
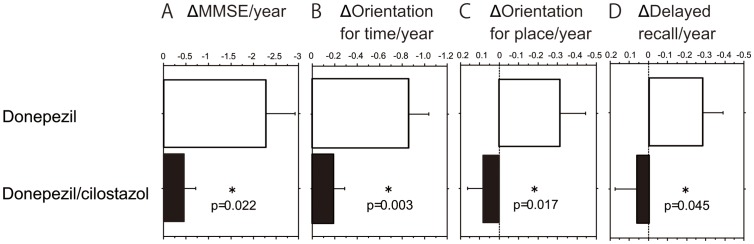
Cilostazol add-on therapy suppressed cognitive decline in patients with mild dementia receiving donepezil. (A) Cilostazol add-on therapy significantly suppressed decrease of total MMSE score (A) and MMSE subscale scores of orientation for time (B), orientation for place (C), and delayed recall (D) in patients with mild dementia receiving donepezil. * p<0.05 versus donepezil group. Error bars show standard error of the mean.

Intriguingly, we observed statistically significant reduced annual change in MMSE in the cilostazol add-on group compared to the donepezil group (−0.5±1.6 versus −2.2±4.1; p = 0.022) (Figure 1). Among the eight variables mentioned above, absence of cilostazol treatment was the only significant predictor of MMSE decline (p = 0.043). The addition of other explanatory variables, such as antihypertensive medications, did not affect the result. The annual change in MMSE subscale scores was significantly different for item 1 (orientation for time; −0.85 vs. −0.16; p = 0.003), item 2 (orientation for place; −0.31 vs. +0.09; p = 0.017), and item 5 (delayed recall; −0.28 vs. +0.05; p = 0.045), thus indicating a beneficial effect of cilostazol on the cognitive domains most vulnerable in AD. These results thus suggested that cilostazol suppresses cognitive decline in patients with mild dementia but not in those with established moderate/severe dementia.

## Discussion

The main finding of this retrospective clinical study is that cilostazol is effective in the preservation of cognitive function for approximately two years in patients with mild dementia receiving donepezil. Multivariate analyses confirmed that only the use of cilostazol was a significant predictor of slower cognitive decline as measured by MMSE among the explanatory variables examined. Intriguingly, the positive effect of cilostazol was found in orientation for time and place and delayed recall, the cognitive domains most vulnerable in early AD.

A large-scale, randomized clinical trial demonstrated a therapeutic effect of cilostazol in the prevention of cerebral vascular disease [15]. In addition to such effects on cerebral circulation, cilostazol is known to reduce accumulation of Aβ and improve brain function in an experimental model of Alzheimer's disease [10]. The experimental study was followed by a pilot study on 10 patients with moderate Alzheimer's disease in a clinical setting where cilostazol slowed the trajectory of cognitive decline when co-administered with donepezil [11]. In addition, cilostazol was found to be effective in halting cognitive decline in patients with AD with cerebrovascular diseases [12] and mild cognitive impairment [13]. In the current study, it is apparent that cerebral circulatory impairment and Aβ accumulation may coexist and vary significantly between patients. However, this situation may be representative of that generally observed in an elderly population, indicating the clinical relevance of administering a drug that has dual roles in ischemia and Aβ-induced neurodegeneration. Thus, the preservation of cognitive function in patients with mild dementia treated with the cilostazol/donepezil combinatorial therapy may represent a finding of important clinical significance.

One of the plausible mechanistic explanations for the positive effect of combinatorial therapy is that donepezil and cilostazol have different vascular targets. Donepezil increases the level of acetylcholine, which in turn dilates vessels in an endothelium-dependent manner, while cilostazol targets PDE3 in the vascular smooth muscle cells and thus causes vasodilation in an endothelium-independent manner. We previously performed preliminary experiments using cerebrovascular β-amyloidosis mice overexpressing mutant human amyloid precursor proteins. Topical treatment with acetylcholine/cilostazol on the brain surface showed a significant increase in the vasodilatory response than that with acetylcholine alone, suggesting that cilostazol restores cerebral hemodynamic reserve in the β-amyloidosis mice (unpublished data). Several ‘single-target, single-action’ treatments for AD, such as anti-amyloid agents, antioxidants, and anti-inflammatory drugs, have mostly failed or performed poorly in large clinical trials [16], leading to the complementary ‘neurovascular hypothesis’ [17]. Multiple pathogenic cascades originating from altered vasculature can initiate disintegration of the neurovascular unit, which can amplify Aβ deposition, synaptic, neuronal and/or glial dysfunction, and subsequent cognitive decline in AD [18]. The current study therefore suggests that the vasoactive cilostazol may be a promising new therapeutic approach to maximize the potential to improve cognitive function in mildly demented patients receiving donepezil.

There are limitations to our retrospective study. First, patients with mild dementia who received cilostazol with donepezil showed a nonsignificant tendency of milder hippocampal atrophy, implying lighter neurodegenerative and heavier ischemic burden despite comparable initial MMSE score in the combinatorial therapy group. In other words, there may have been an intergroup difference in the patient demographics as a result of patient selection bias. The reasons why some patients but not others received cilostazol were unclear, which is an inherent limitation of such retrospective analyses. Second, the three major cardiovascular risk factors were more frequent in donepezil group compared to the cilostazol add-on group. This suggests an intergroup difference in background factors; however, a higher frequency of cardiovascular risk factors in the donepezil alone group may suggest a heavier ischemic burden in this group, a trend opposite to that mentioned in the first limitation. Third, we only evaluated cognitive function based on the MMSE, where multidimensional tests would have been preferable in order to evaluate cognitive function more accurately. Therefore, diagnosis of the clinical status of enrolled patients may not be sufficiently specific to allow conclusions regarding specific types of dementia. Finally, the sample size of this study is rather small; thus, comprehensive testing with a larger sample is required.

Regardless of such limitations, our results show that cognitive function can be maintained, even in a heterogeneous population with mild dementia, with cilostazol, affecting both cerebral circulation and Aβ metabolism. Since there is no fundamental treatment for dementia, development of preventive therapy is eagerly awaited. Our results highlight the need for a comprehensive prospective cohort study to analyze the effect of cilostazol on the preservation of cognitive function in patients with early-stage cognitive impairment.

## References

[1] Gorelick PB, Scuteri A, Black SE, Decarli C, Greenberg SM, et al (2011) Vascular contributions to cognitive impairment and dementia: a statement for healthcare professionals from the American Heart Association/American Stroke Association. Stroke 42: 2672–2713. 21778438 10.1161/STR.0b013e3182299496 PMC3778669

[2] Toledo JB, Arnold SE, Raible K, Brettschneider J, Xie SX, et al (2013) Contribution of cerebrovascular disease in autopsy confirmed neurodegenerative disease cases in the National Alzheimer's Coordinating Centre. Brain 136: 2697–2706. 23842566 10.1093/brain/awt188 PMC3858112

[3] Kalaria RN, Akinyemi R, Ihara M (2012) Does vascular pathology contribute to Alzheimer changes? J Neurol Sci 322: 141–147. 22884479 10.1016/j.jns.2012.07.032

[4] Kalaria RN, Ihara M (2013) Dementia: Vascular and neurodegenerative pathways-will they meet? Nat Rev Neurol. 487–488. 10.1038/nrneurol.2013.164 23938746

[5] Liu Y, Shakur Y, Yoshitake M, Kambayashi JJ (2001) Cilostazol (pletal): a dual inhibitor of cyclic nucleotide phosphodiesterase type 3 and adenosine uptake. Cardiovasc Drug Rev 19: 369–386. 11830753 10.1111/j.1527-3466.2001.tb00076.x

[6] Kim KY, Shin HK, Choi JM, Hong KW (2002) Inhibition of lipopolysaccharide-induced apoptosis by cilostazol in human umbilical vein endothelial cells. J Pharmacol Exp Ther 300: 709–715. 11805237 10.1124/jpet.300.2.709

[7] Fujita Y, Lin JX, Takahashi R, Tomimoto H (2008) Cilostazol alleviates cerebral small-vessel pathology and white-matter lesions in stroke-prone spontaneously hypertensive rats. Brain Res 1203: 170–176. 18321473 10.1016/j.brainres.2008.01.103

[8] Tanaka K, Gotoh F, Fukuuchi Y, Amano T, Uematsu D, et al (1989) Effects of a selective inhibitor of cyclic AMP phosphodiesterase on the pial microcirculation in feline cerebral ischemia. Stroke 20: 668–673. 2718208 10.1161/01.str.20.5.668

[9] Hiramatsu M, Takiguchi O, Nishiyama A, Mori H (2010) Cilostazol prevents amyloid β peptide(25–35)-induced memory impairment and oxidative stress in mice. Br J Pharmacol 161: 1899–1912. 20825411 10.1111/j.1476-5381.2010.01014.x PMC3010591

[10] Park SH, Kim JH, Bae SS, Hong KW, Lee D-S, et al (2011) Protective effect of the phosphodiesterase III inhibitor cilostazol on amyloid β-induced cognitive deficits associated with decreased amyloid β accumulation. Biochem Biophys Res Commun 408: 602–608. 21530492 10.1016/j.bbrc.2011.04.068

[11] Arai H, Takahashi T (2009) A combination therapy of donepezil and cilostazol for patients with moderate Alzheimer disease: pilot follow-up study. Am J Geriatr Psychiatry 17: 353–354. 19307864 10.1097/JGP.0b013e31819431ea

[12] Sakurai H, Hanyu H, Sato T, Kume K, Hirao K, et al (2013) Effects of cilostazol on cognition and regional cerebral blood flow in patients with Alzheimer's disease and cerebrovascular disease: a pilot study. Geriatr Gerontol Int 13: 90–97. 22672107 10.1111/j.1447-0594.2012.00866.x

[13] Taguchi A, Takata Y, Ihara M, Kasahara Y, Tsuji M, et al (2013) Cilostazol improves cognitive function in patients with mild cognitive impairment: A retrospective analysis. Psychogeriatrics 13: 164–169. 25707423 10.1111/psyg.12021

[14] Kamiyama K, Wada A, Sugihara M, Kurioka S, Hayashi K, et al (2010) Potential hippocampal region atrophy in diabetes mellitus type 2: a voxel-based morphometry VSRAD study. Jpn J Radiol 28: 266–272. 20512543 10.1007/s11604-009-0416-2

[15] Shinohara Y, Katayama Y, Uchiyama S, Yamaguchi T, Handa S, et al (2010) Cilostazol for prevention of secondary stroke (CSPS 2): an aspirin-controlled, double-blind, randomised non-inferiority trial. Lancet Neurol 9: 959–968. 20833591 10.1016/S1474-4422(10)70198-8

[16] Zlokovic BV (2011) Neurovascular pathways to neurodegeneration in Alzheimer's disease and other disorders. Nat Rev Neurosci 12: 723–738. 22048062 10.1038/nrn3114 PMC4036520

[17] Chow N, Bell RD, Deane R, Streb JW, Chen J, et al (2007) Serum response factor and myocardin mediate arterial hypercontractility and cerebral blood flow dysregulation in Alzheimer's phenotype. Proc Natl Acad Sci U S A 104: 823–828. 17215356 10.1073/pnas.0608251104 PMC1783398

[18] Weller RO, Djuanda E, Yow HY, Carare RO (2009) Lymphatic drainage of the brain and the pathophysiology of neurological disease. Acta Neuropathol 117: 1–14. 19002474 10.1007/s00401-008-0457-0

